# RIP3 knockdown inhibits necroptosis of human intestinal epithelial cells via TLR4/MyD88/NF-κB signaling and ameliorates murine colitis

**DOI:** 10.1186/s12876-022-02208-x

**Published:** 2022-03-26

**Authors:** Chaoqin Duan, Xi Xu, Xiaoyi Lu, Ling Wang, Zhongkai Lu

**Affiliations:** Department of Gastroenterology, Suzhou Municipal Hospital, Suzhou Hospital Affiliated to Nanjing Medical University, 16 Baita West Road, Suzhou, 215001 Jiangsu Province China

**Keywords:** Ulcerative colitis, Nec-1, Necroptosis, Receptor interacting protein kinase 3, TLR4-MyD88

## Abstract

**Background:**

Ulcerative colitis (UC) is a common inflammatory bowel disease, during which cell necroptosis plays key roles in driving inflammation initiation and aggravation. Previous studies reported Receptor Interacting Protein Kinase 3 (RIP3)-mediated necroptosis in multiple diseases, and RIP3 protein in Paneth cells significantly enriched in the intestines of both humans and mice. Therefore, we hypothesized targeting RIP3 to inhibit necroptosis may depress UC.

**Methods:**

We classified clinical UC samples according to the modified Truelove & Witts criterion. The expression of RIP3 was measured by western blot and immunohistochemistry. Cell proliferation and apoptosis were analyzed by MTT assay and flow cytometry. ROS production and the secretion of inflammatory cytokines were measured by DCFH-DA probe and ELISA assay. TLR4/MyD88/NF-κB signaling pathway was analyzed by western blot. We established experimental colitis model in RIP3 knockout and wild-type mice and disease activity index (DAI) score was calculated. The expression and distribution of tight junction protein were analyzed by immunofluorescence. The ratio of CD4^+^Foxp3^+^ T cells in the spleen was detected by flow cytometry. Oxidative damage of mouse colon was assessed by detecting the levels of SOD, MDA and MPO. Data were analyzed by one-way ANOVA or student’s *t* test.

**Results:**

The expression of RIP3 in human colon is positively associated with the severity of UC. RIP3 inhibitor GSK872 or RIP3 knockdown reverses the inhibitory effect of TNF-α on proliferation and the promoting effect of TNF-α on apoptosis and necrosis in human intestinal epithelial cells. In addition, RIP3 deficiency inhibits the secretion of inflammatory cytokines (IL-16, IL-17 and IFN-γ) and ROS production induced by TNF-α. In vivo, RIP3 inhibitor Nec-1 effectively improves DSS-induced colitis in mice. In mechanism, RIP3 depression could upregulate the proportion of CD4^+^Foxp3^+^ immunosuppressive Treg cells in the spleen while suppressed TLR4/MyD88/NF-κB signaling pathway and ROS generation, and all these anti-inflammation factors together suppress the secretion of inflammatory cytokines and necroptosis of intestinal epithelial cells.

**Conclusions:**

This study preliminarily explored the regulating mechanism of RIP3 on UC, and Nec-1 may be a promising drug to alleviate the inflammation and necroptosis of the colon in UC patients.

**Supplementary Information:**

The online version contains supplementary material available at 10.1186/s12876-022-02208-x.

## Background

Ulcerative colitis (UC) is severe inflammatory bowel disease, characterized by non-specific inflammation in intestinal tract. UC occurrence often accompanied by intestinal mucosal necrosis and shedding, ulcer formation and excessive proliferation, and even cancerous transformation, which seriously affects the life quality of UC patients [1].

Necroptosis is a typical form of regulatory necrosis triggered by death receptors induced by tumor necrosis factor-α (TNF-α) [2]. Unlike apoptosis characterized by cell shrinkage, nucleus rupture and apoptotic body formation, necroptosis involves cell membrane rupture and requires the formation of various protein complexes which are called necrosomes [3]. Evidence showed that the interaction of receptor interacting protein kinase 1 (RIP1) and RIP3 leads to RIP3–RIP3 homo-interaction and RIP3 autophosphorylation. Phosphorylated RIP3 recruits and phosphorylates MLKL, which then is translocated to the cell membrane to perform necrotizing lesions [3]. Although the initial molecular components of the necrotic pathway have been described, the downstream signal regulation mechanism is still not very clear.

TNF-induced necroptosis is related to energy metabolism. After TNF stimulation, the regulatory NADPH oxidase organizer 1 (NOXO1) subunit associates with RIP1, TRADD and Rac, and initiates the production of ROS through the cell membrane-associated NADPH oxidase NOX1. These ROS cause a prolonged activation time of JNK, leading to TNF-induced necroptosis [4]. However, whether RIP3 is also involved in this type of TNF-induced ROS production has not been explored. Generally, TLR4-MyD88 signaling pathway is closely correlated with the inflammatory signaling pathway activation during UC. When pro-inflammation signal is transferred from TLR4 to MyD88, with the continuous recruitment of IRAK4 and TRAF6, the IKK complex is activated, causing the proteasome to destroy IκB. Subsequently, NF-κBp65 subunit is translocated into the nucleus and promotes the production of pro-inflammatory cytokines, accelerating the progression of inflammation [5].TNF-α involves the initiation of inflammation in the intestinal mucosa of UCs. Currently, TNF-α monoclonal antibody (infliximab) is used clinically to treat UC and has achieved good efficacy, but there are also side effects. Recent studies have found that RIP3 is the key effector of the TNF-α signaling pathway, and becomes the bifurcation point of TNF-α-mediated pro-apoptosis and pro-necrosis [6]. We speculate whether the development of antagonists of TNF-α downstream effectors that regulate cell necroptosis can replace TNF-α monoclonal antibody in the treatment of UC?

It was rarely reported whether RIP3 was involved in the onset and course of UC. Studies have shown that there were a large amount of RIP3 protein enrichment in both human and mouse intestinal Paneth cells. In the TNF-α-induced colitis mouse model, the intestinal mucosa of wild-type mice is massively necrotic, while RIP3^−/−^ mice do not have this phenomenon [7]. This indicates that RIP3 may play an important role in mediating intestinal mucosal inflammation.

Since the discovery of a small chemical molecule (Nec-1) inhibiting programmed necrosis in 2005, scholars have explored the value of Nec-1 in the treatment of diseases. Nec-1 is a specific cell necroptosis inhibitor, which can directly inhibit the expression of RIP3, which, therefore, may have a therapeutic effect on necroptosis-related diseases. Studies have shown that Nec-1 can effectively inhibit autophagy and apoptosis in mice with traumatic brain injury. Studies have also shown that Nec-1 can alleviate the dysfunction of neuron and astrocyte mitochondria in ischemic mice [8, 9], and promote long-term functional recovery by protecting oligodendrocyte precursor cells after focal cerebral ischemia [10]. Therefore, whether Nec-1 can alleviate the inflammation and necrotic cell death of the colon in patients with UC is remind to be explored.

This study explored the effects of RIP3 inhibitors on the proliferation, apoptosis, necrosis, inflammatory cytokine secretion and ROS production of human intestinal epithelial cells induced by TNF-α in vitro. Then, we demonstrated the therapeutic effect of Nec-1 and RIP3 knockdown on DSS-induced colitis mice. This study suggested that Nec-1 and RIP3 suppression may be a promising therapeutic strategy to alleviate the inflammation and necroptosis of the colon in patients with UC.

## Materials and methods

### Cell culture and transfection

Normal human intestinal epithelial cells (HIEC-6, ATCC® CRL-3266) were cultivated in RPMI-1640 medium (Gibco, CA, USA) with 10% fetal bovine serum (16000-044, Gibco, CA, USA). Cells were seeded in a six-well plate and incubated in incubator at 37 °C with 5% carbon dioxide (CO2) and 95% air. When density reached about 70%, plasmids were transfected according to the instruction of Lipofectamine™ 3000 (Thermo Fisher Scientific, MA, USA). RNA was extracted after 48 h, and protein was after 72 h. The mass of plasmid transfected in each well is 800 ng.

### Construction of RIP3 knockdown vector

Primers were designed according to the Sigma-Aldrich website (http://rnaidesigner.thermofisher.com/rnaiexpress/) and the pSilencer2.1_U6 vector instructions. The sequences for shRNA were as follows: F: GATCCGCAAGTCTGGATAACGAATTCTTCAAGAGAGAATTCGTTATCCAGACTTGCTTTTTTGGAAA.

R:AGCTTTTCCAAAAAAGCAAGTCTGGATAACGAATTCTCTCTTGAAGAATTCGTTATCCAGACTTGCG. The pSilencer2.1-U6 neo vector was purchased from Invitrogen. The primers were annealed according to the instructions of Annealing Buffer for DNA Oligos (Shanghai Biyuntian Biotechnology Co., Ltd.). Procedures are as follows: 95 °C, 2 min; decrease by 0.1 °C every 8 s to 25 °C, ~ 90 min; 4 °C, ∞. pSilence2.1-U6 neo vector was digested with HindIII and BamHI; enzyme linked overnight at 16 °C; enzyme linked product was recombined, transformed and plated. After the colony is amplified, plasmids are extracted and sent to BGI for sequencing. The universal primer for sequencing is U6-promoter-human (GGACTATCATATGCTTACCG).

### Western blot

The experimental procedures were performed as previously reported [2]. The extraction of total protein was performed on ice throughout the entire process. The protein concentration was determined by Bradford method. Total proteins were separated on a 10% SDS-PAGE gel, then transferred onto PVDF membranes (Bio-Rad Laboratories Inc., CA, USA). After blocked with 5% non-fat milk for 1 h, membranes were incubated with antibodies RIP3 (ab62344, Abcam, Cambridge, UK), MLKL (ab184718, Abcam), MyD88 (ab219413, Abcam), TLR4 (ab13556, Abcam), p-IκB (CY6280, Abways Technology Inc., Shanghai, China), IκB (CY2327, Abways) and p65 (ab32536, Abcam). β-actin (AB0011, Abways) or histone H3 (ab1791, Abcam) was used as the loading control. Then, membranes were incubated with Peroxidase AffiniPure Goat Anti-Rabbit IgG (H + L) (111-035-003, 1:10,000, Jackson ImmunoResearch Inc., PA, USA) for 1 h and then HRP chemiluminescence detection reagent (Tiangen Biotech, Beijing, China). Tanon 5200 automatic chemiluminescence image analysis system (Tanon, Beijing, China) was applied for ECL imaging. Image J software (Rawak Software Inc., Stuttgart, Germany) performed semi-quantitative analysis.

### Immunohistochemistry

Mucosal biopsies were collected from 20 patients with UC undergoing colonoscopy or surgery at the Suzhou Hospital Affiliated to Nanjing Medical University between March 2021 and July 2021. Informed consent was obtained from all the patients, and the hospital ethics committees approved this study. Clinical disease activity was evaluated using a modified Truelove–Witts severity index [11]. Clinical characteristics of included ulcerative colitis patients have been shown in Table 1. The experimental procedures were performed as previously reported [12]. After fixing the tissues with 4% paraformaldehyde at 4 °C for 24 h, the sections were embedded in paraffin, and then the IHC steps were as follows: dewaxing in xylene and ethanol; preheating sodium citrate (pH = 6.0) antigen retrieval solution at high temperature for 10 min, the tissue section is boiled in the solution for 30 min; 3% H2O2 for 10 min; 3% BSA for 1 h. Immunohistochemical staining of paraffin-embedded tissue was conducted. RIP3 antibody (ab62344, Abcam), MLKL antibody (ab184718, Abcam) and CD68 antibody (ab125212, Abcam) were diluted with PBS buffer at a ratio of 1:100. The slides were incubated with antibodies overnight at 4 °C and then DAB solution for 3 min; hematoxylin counterstain for 2 min; 1% hydrochloric acid ethanol differentiation for 2 s; dehydrate with ethanol and xylene. After the xylene was drained, neutral gum was added to mount the slide. Staining results were examined under microscope with 20 × objective. Eight fields of view were randomly selected under microscope. Image-Pro Plus 6.0 software (Rawak Software Inc., Stuttgart, Germany) was used for semi-quantitative analysis.

**Table 1:** Clinical characteristics of included ulcerative colitis patients

Variables	Number (n = 20)
Sex (male/female)	13/7
Age (years)	45.50 ± 10.76
*Smoking status*	
Smoking	7
Non-smoking	13
*Disease activity adapted from Truelove and Witts*	
Mild	5
Moderate	7
Severe	8
*Medication*	
5-ASA	6
Immunomodulators or biologics	6
Glucocorticoids	8

### Real-time RT-PCR

The experimental procedures were performed as previously reported [13]. Total RNA was extracted by Trizol (Invitrogen, CA, USA). cDNA was synthesized by Hifair II 1st Strand cDNA Synthesis SuperMix (Yeasen, Shanghai, China). Follow the instructions of Hieff UNICON® qPCR SYBR Green Master Mix (Yeasen, Shanghai, China) to configure the Real-Time PCR reaction system and run qPCR program: step1—95 °C, 30 s; step2—95 °C, 5 s; step3—60 °C, 20 s; step4—72 °C, 20 s; step1–3 repeat 40 cycles. GAPDH was used as a housekeeping gene. The sequences of specific primers are as follows. GAPDH-F: CCTTCCGTGTCCCCACT, GAPDH-R: GCCTGCTTCACCACCTTC; RIP3-F: TCCAGGGAGGTCAAGGC; RIP3-R: ACAAGGAGCCGTTCTCCA. The expression level of each group of genes was analyzed using 2^−ΔΔCT^.

### Cell viability detection

Human intestinal epithelial cells were treated with TNF-α (10 ng/ml, 12 h) (96-300-01A-10, PeproTech) and then shRIP3 plasmid or 2 μM RIP3 inhibitor GSK-872 (CAS No. 1346546-69-7, MedChemExpress) for 24 h. 20 μl of 5 mg/ml MTT was added to each well. After 4 h of treatment with MTT (Beyotime Biotech, Shanghai, China), the supernatant was discarded, and 100μL of dimethyl sulfoxide (DMSO) was added. Optical density value (OD value) was detected at wavelengths of 492 nm and 630 nm, and the cell survival rate was calculated according to the formula: cell survival rate (%) = [experimental group OD average value-background group OD average value]/[Blank control group OD average value-background group OD average value] × 100%

### Flow cytometry analysis of apoptosis

1 × 10^6^ human intestinal epithelial cells were cultured in a six-well plate and treated with TNF-α and then shRIP3 plasmid or RIP3 inhibitor GSK-872 for 24 h. The cells were incubated with 5 μl V-FITC and 5 μl PI (BD Biosciences Pharmingen, CA, USA) for 15 min in the dark and then resuspend in 400 μl 1 × binding buffer. The proportion of apoptotic cells was analyzed by flow cytometer (Becton, Dickinson and Company, NJ, USA). FlowJo 7.6 software was applied for data analysis.

### Immunofluorescence histochemistry

The experimental procedures were performed as previously reported [5]. Slices were prepared from 4% paraformaldehyde-fixed and paraffin-embedded mice colon tissues.

The steps of dewaxing and antigen retrieval was the same as those of immunohistochemistry. The sections were blocked with 3% BSA-PBS at room temperature for 1 h and incubated with anti-mouse antibody against ZO-1 (ab221547, Abcam, dilute at 1:100) overnight at 4 °C, then secondary antibody goat anti-rabbit IgG (H + L) with FITC fluorescent label (Jackson ImmunoResearch Inc., PA, USA, dilute at 1:100) at room temperature for 1 h. Nuclei was stained with 1 μg/ml DAPI (Sigma-Aldrich, MO, USA) for 10 min. Neutral gum is added to mount the slide. Slices were examined under fluorescence microscope with a 20 × objective.

### ELISA assay

The supernatant of the cells was collected and centrifuged at 4 °C and 10,000 rpm for 5 min. Small sections (~ 1 cm) of excised distal colonic tissue were collected. Then, ~ 10 mg colon tissue was homogenized with 1 ml PBS and centrifuged at 16,000*g* for 20 min at 4 °C. The levels of IL-6 (ab178013, Abcam), IL-17 (ab100556, Abcam), IFN-γ (ab174443, Abcam), IL-10 (cat. no. EK0417, Boster Biological Technology), SOD (BC0170, Solarbio, Beijing, China), MDA (BC0020, Solarbio) and MPO (ab105136, Abcam) in the supernatant were detected according to the product instructions.

### ROS generation detection

2 × 10^5^ human intestinal epithelial cells were cultured in a six-well plate. A fluorescent probe 10 μM DCFH-DA (37 °C, 20 min) was used to detect the level of intracellular ROS. Eight fields of view were randomly photographed under fluorescence microscope.

### Animal model [14]

A total of 20 6-week-old male BALB/c mice were purchased from Jie Si Jie Laboratory, Shanghai, China. Five RIP3 knockout mice [15] (C57BL/6N-Ripk3em1) was purchased from Cyagen, Guangzhou, China. The animal research was approved by the Ethics Committee of Nanjing Medical University (approval no. SYXK(S) 2020-0022). Mice are kept in a sterile environment with constant temperature and humidity, and are allowed to eat and drink freely. Mice were randomly divided into five groups (n = 5 per group). The experimental colitis model was established by 3% (w/v) DSS (Sigma-Aldrich; Merck KGaA) in drinking water for 7 days. On the 8th day, mice were intraperitoneally administered with Necrostatin-1 (Nec-1, a specific cell necroptosis inhibitor) (MedChemExpress, CAS No. 4311-88-0, 5 mg/kg), SASP (Merck KGaA, cat. no. 599-79-1, 50 mg/kg) and 1% DMSO, respectively, for 14 consecutive days. Mice were euthanized with an intraperitoneal injection of 150 mg/kg sodium pentobarbital, and then the heartbeat of the mice was observed. The duration of animal experiments was 2 weeks from the first day of treatment with DSS to sac.

### Mouse disease activity index and colonic mucosal damage index

Mice of each group were examined daily for body mass, diarrhea and blood in the stool. Disease activity index (DAI) = (weight loss score + fecal trait score + fecal occult blood score)/3. Colonic mucosal damage index (CMDI) was scored according to the criteria [16].

### Hematoxylin and eosin (H&E) staining

Distal colon tissue was fixed in 4% paraformaldehyde for 24 h at 4 °C, and then embedded in paraffin, sectioned to a thickness of 8 µm. The sections were subjected to the following steps: xylene dewaxing; gradient ethanol hydration; hematoxylin staining for 5 min; water washing for 5 min; 1% hydrochloric acid ethanol differentiation for 5 s; weak alkaline aqueous solution for 3 s; eosin staining for 5 min; gradient ethanol dehydration; Mounting with neutral gum. Histopathological changes were observed under a light microscope (magnification, 20 ×) and scored according to the criteria described by Andújar et al. [17]. The colon histopathology index (CHPI) was determined as previously described [18].

### Flow cytometry analysis

The whole spleen is separated, cut into pieces, and filtered through a 200-mesh sieve. The suspend mouse spleen cells were washed and adjusted to 1 × 10^6^/ml and blocked with 2.5% BSA for 1 h and then incubated with FITC-rat anti-mouse CD4 (catalog no RM4-5; eBioscience; Thermo Fisher Scientific, Inc.) for 0.5 h in the dark and fixed with Transcription Factor Staining Buffer Set (catalog no. 00-5523-00; eBioscience; Thermo Fisher Scientific, Inc.) for 15 min. Cells were then incubated with PE-rat anti-mouse Foxp3 (catalog no. 72-5775-40; eBioscience; Thermo Fisher Scientific, Inc.) for 1 h at 4 °C in the dark and detected by CytoFLEX V0-B5-R3 Flow Cytometer (Life Sciences, Indianapolis, Indiana).

### Statistical analysis

The data were analyzed by Graphpad Prism 8.0 (GraphPad Software Inc., CA, USA), and expressed as Mean ± standard deviation (SD). For in vitro experiments, we repeated three times, each containing at least three replicate samples. Results comparing multiple groups were analyzed by one-way ANOVA followed by Tukey’s post hoc test. Results comparing two groups were analyzed by student’s *t* test. Difference among groups was considered significant when *P* value < 0.05.

## Results

### The expression of RIP3 in the colon is positively proportional to the severity of UC

**Fig. 1:**
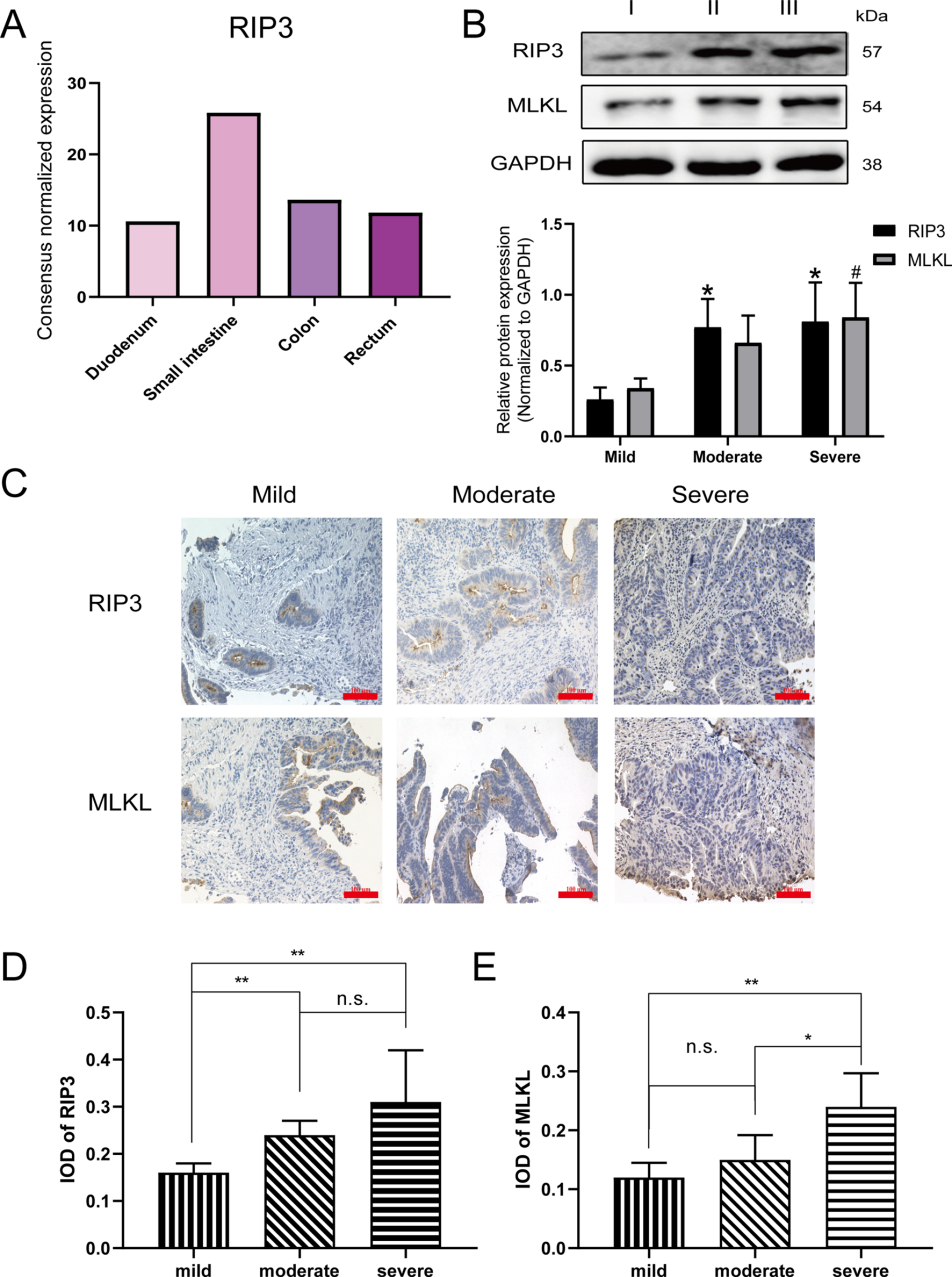
The expression of RIP3 in the colon is positively proportional to the severity of UC. **A** The Human Protein Atlas (https://www.proteinatlas.org/search/RIPK3) analyzed RNA expression of RIP3 in more than 60 human tissues and organs. Interestingly, among more than 60 human tissues and organs, RIP3 has the most RNA expression in small intestines. We have shown the RNA expression of RIP3 in Duodenum, Small intestine, Colon, and Rectum. **B** Western blot detected the expression levels of RIP3 and MLKL in the colon tissues from patients with mild (I), moderate (II) and severe (III) UC based on the modified Truelove & Witts severity classification. The semi-quantitative analysis of western blotting via Image J software. RIP3: *P < 0.05, ~ versus Mild; MLKL: ^#^*P* < 0.05, ~ versus Mild. **C** Immunohistochemistry detected the expression and distribution of RIP3 and MLKL in the colon tissues from patients with mild, moderate and severe UC. **D**–**E** The mean optical density value (IOD) of the target protein in each field was analyzed by Image-Pro Plus 6.0 software. Scale bar: 100 μm. The data were presented as mean ± SD. The asterisk indicates a significant difference (*P < 0.05, **P < 0.01) between groups. Abbreviations: n.s., no significance

Necroptosis is a novel regulatory necrosis pathway that requires receptor-interacting protein kinase 3 (RIP3) and mixed-lineage kinase domain-like protein (MLKL). It has been shown that changes in the expression of RIP3 and MLKL involved in excessive death of epithelial cells by a manner of necroptosis, thereby triggering intestinal inflammation. MLKL was the target of RIP3, acts downstream of RIP1 and RIP3, and is recruited into the necrosome through interaction with RIP3 [19]. We first applied The Human Protein Atlas (https://www.proteinatlas.org/search/RIPK3) to observe the RNA expression of RIP3 in more than 60 human tissues and organs. We compared the RNA expression of RIP3 in duodenum, small intestine, colon, and rectum (Fig. 1A). Interestingly, among more than 60 human tissues and organs, RIP3 has the highest RNA expression in small intestines.

Subsequently, we collected clinical colon tissues from patients with mild, moderate, and severe UC according to the criterion of the modified Truelove & Witts severity classification [11]. Western blot results showed that the expression levels of RIP3 and MLKL significantly increased with the severity of UC increased. (Fig. 1B). Immunochemistry assay show that RIP3 was mainly distributed in the cytoplasm in the intestinal tissue of patients with mild and moderate UC, while RIP3 was mainly distributed in the cytoplasm and nucleus in the intestinal tissue of patients with severe UC (Fig. 1C). IHC semi-quantitative analysis showed that the expression level of RIP3 in the intestinal tissue of severe UC patients was higher than that in moderate UC patients (*P* < 0.01) (Fig. 1D). Compared with mild and moderate UC, the expression of MLKL in the intestinal tissue of patients with severe UC was higher (*P* < 0.05). (Fig. 1D).

### Knockdown of RIP3 expression inhibits the pro-apoptotic effect of TNF-α on intestinal epithelial cells

**Fig. 2:**
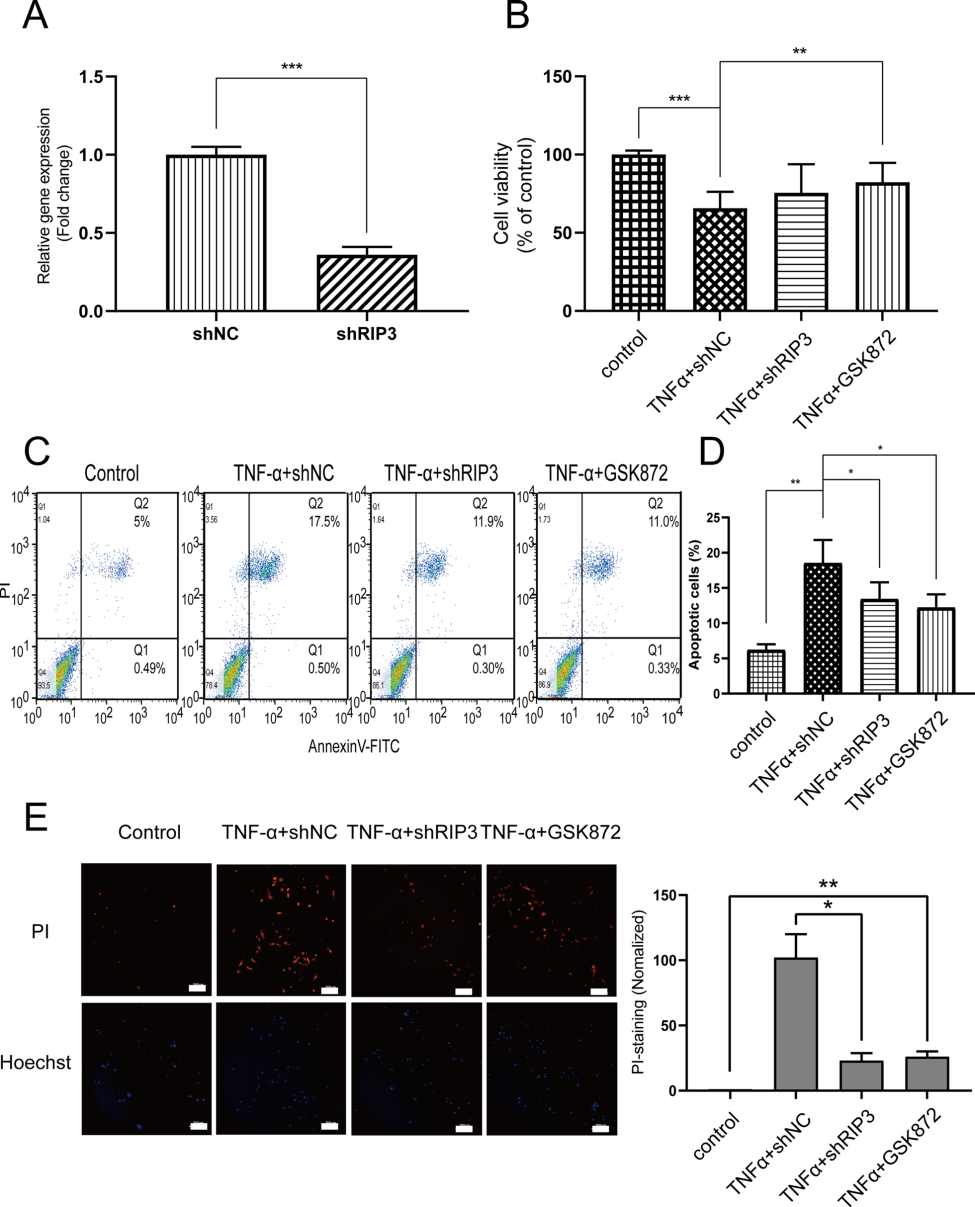
Knockdown of RIP3 expression inhibits the pro-apoptotic effect of TNF-α on intestinal epithelial cells. **A** RT-qPCR demonstrated the knockdown efficiency of shRIP3 plasmid. **B** MTT assay detected the effect of shRIP3 knockdown plasmid and RIP3 inhibitor GSK872 (2 μM) on cell viability after TNF-α (10 ng/ml, 12 h) treatment. **C** Annexin V-FITC and PI double staining detected the effect of shRIP3 knockdown plasmid and RIP3 inhibitor GSK872 on cell apoptosis. **D** Semi-quantitative analysis of cell apoptosis (The sum of the percentages of the first and fourth quadrants). **E** PI staining detected cell necrosis and the results were observed under fluorescence microscope with a 20 × objective. Blue color: Hochest 33342 stains the nucleus; Green color: PI Dye. Scale bar: 200 μm. The semi-quantitative analysis of immunofluoresence via Image J software (right panel)

TNF-α can induce either apoptosis or necroptosis in different cell lines [3]. RIP3 is involved in NF-κB-regulated programmed cell death, including apoptosis and necroptosis. RIP3 interacts with RIP1 with RIP homotypic interaction motif and then promotes apoptosis by activating caspases and/or inhibiting TNFR1-induced NF-κB activation [20, 21]. To investigate the effect of RIP3 knockdown on the survival of intestinal epithelia cells, we constructed the shRIP3 knockdown cell line in vitro. RT-qPCR verified that it could effectively reduce the mRNA level of RIP3 in normal human intestinal epithelial cells (Fig. 2A). Subsequently, MTT assay detected the effect of shRIP3 knockdown and RIP3 inhibitor GSK872 on cell viability after TNF-α (10 ng/ml, 12 h) induction. The results showed that TNF-α significantly inhibited cell proliferation (*P* < 0.001) (Fig. 2B). In contrast, RIP3 knockdown and GSK872 could significantly rescue the proliferation inhibitory effect of TNF-α on intestinal epithelial cells (*P* < 0.01) (Fig. 2B).

Then, we used Annexin V-FITC and PI double staining to detect the effects of shRIP3 knockdown and GSK872 on cell apoptosis. Flow cytometry results showed that TNF-α enhanced the proportion of apoptotic cells (*P* < 0.01) while RIP3 knockdown and GSK872 treatment significantly reversed the apoptosis-inducing effect of TNF-α on intestinal epithelial cells (*P* < 0.05) (Fig. 2C–D). Next, we observed PI staining results with a fluorescence microscope and found that TNF-α promoted red fluorescence intensity, and RIP3 knockdown and GSK872 inhibited red fluorescence intensity (Fig. 2E). Taken together, RIP3 inhibition could rescue TNF-α-induced apoptosis in normal human intestinal epithelial cells.

### Knockdown of RIP3 expression inhibits inflammatory signaling pathways in vitro

**Fig. 3:**
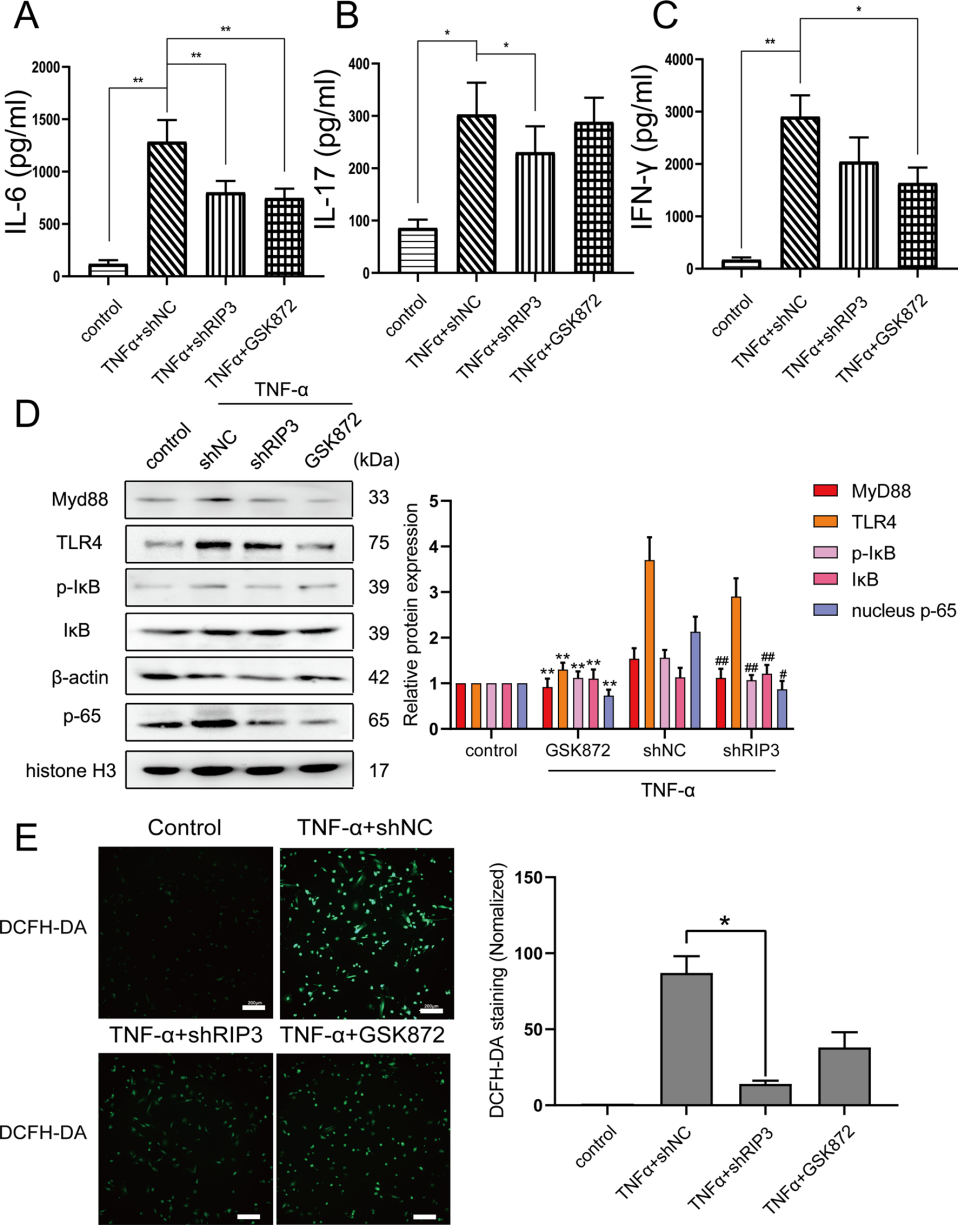
Knockdown of RIP3 expression inhibits inflammatory signaling pathways in vitro. **A**–**C** ELISA assay detected the effect of RIP3 knockdown plasmid and RIP3 inhibitor GSK872 on the secretion of inflammatory factors IL-16, IL-17 and IFN-γ after TNF-α (10 ng/ml, 12 h) treatment. **D** Western blot detected the effect of shRIP3 knockdown plasmid and RIP3 inhibitor GSK872 on the expression of key molecules in the TLR4/MyD88/NF-κB signaling pathway. The semi-quantitative analysis of western blotting via Image J software (right panel). **E** After cultured with TNF-α (10 ng/ml, 12 h), shRIP3 plasmid, or RIP3 inhibitor GSK872, cells were incubated with DAFH-DA probe and the results were observed under fluorescence microscope with a 10 × objective. Scale bar: 200 μm. The semi-quantitative analysis of immunofluoresence via Image J software (right panel)

To explore the effect of RIP3 on activation of inflammatory cytokines, we performed ELISA to detect the effect of shRIP3 knockdown and GSK872 on the secretion of inflammatory factors IL-16, IL-17 and IFN-γ after TNF-α (10 ng/ml, 12 h) treatment. We observed that TNF-α significantly induced the secretion of IL-16, IL-17 and IFN-γ (*P* < 0.05) (Fig. 3A–C). RIP3 knockdown plasmid could inhibit the secretion of IL-6 and IL-17 (*P* < 0.05), and RIP3 inhibitor GSK872 inhibited the secretion of IL-6 and IFN-γ (*P* < 0.05) (Fig. 3A–C). It was reported that RIP3 could promote apoptosis by inhibiting NF-κB activation [21]. However, the influence of RIP3 on NF-κB activation remains controversial. Subsequently, we used western blot to detect the effects of RIP3 knockdown and GSK872 on the expression of key molecules in the TLR4/MyD88/NF-κB signaling pathway. Experimental results showed that TNF-α (10 ng/ml, 12 h) significantly promoted the expression of Myd88, TLR4, p-IκB, and p-65 in intestinal epithelial cells. RIP3 plasmid and GSK872 suppressed the expression of IL-16, IL-17 and IFN-γ induced by TNF-α (Fig. 3D). Under normal physiological conditions, cells control ROS levels by balancing the production and elimination of ROS. Under oxidative stress conditions, excessive ROS can destroy cellular proteins, lipids and DNA, leading to cellular carcinogenesis [22]. Next, we explored whether the pro-apoptotic effect of RIP3 was related to the regulation of intracellular ROS levels. After treating the cells with TNF-α (10 ng/ml, 12 h), RIP3 knockdown and GSK872, we incubated the cells with DAFH-DA probe. We observed under fluorescence microscope that TNF-α could obviously induce intracellular ROS accumulation compared with the blank control group. Compared with the TNF-α control group, RIP3 knockdown and GSK872 significantly inhibited ROS production (Fig. 3E).

### Nec-1, a RIP3 inhibitor, treatment relieves intestinal pathology and histopathology in DSS-induced colitis mice

**Fig. 4:**
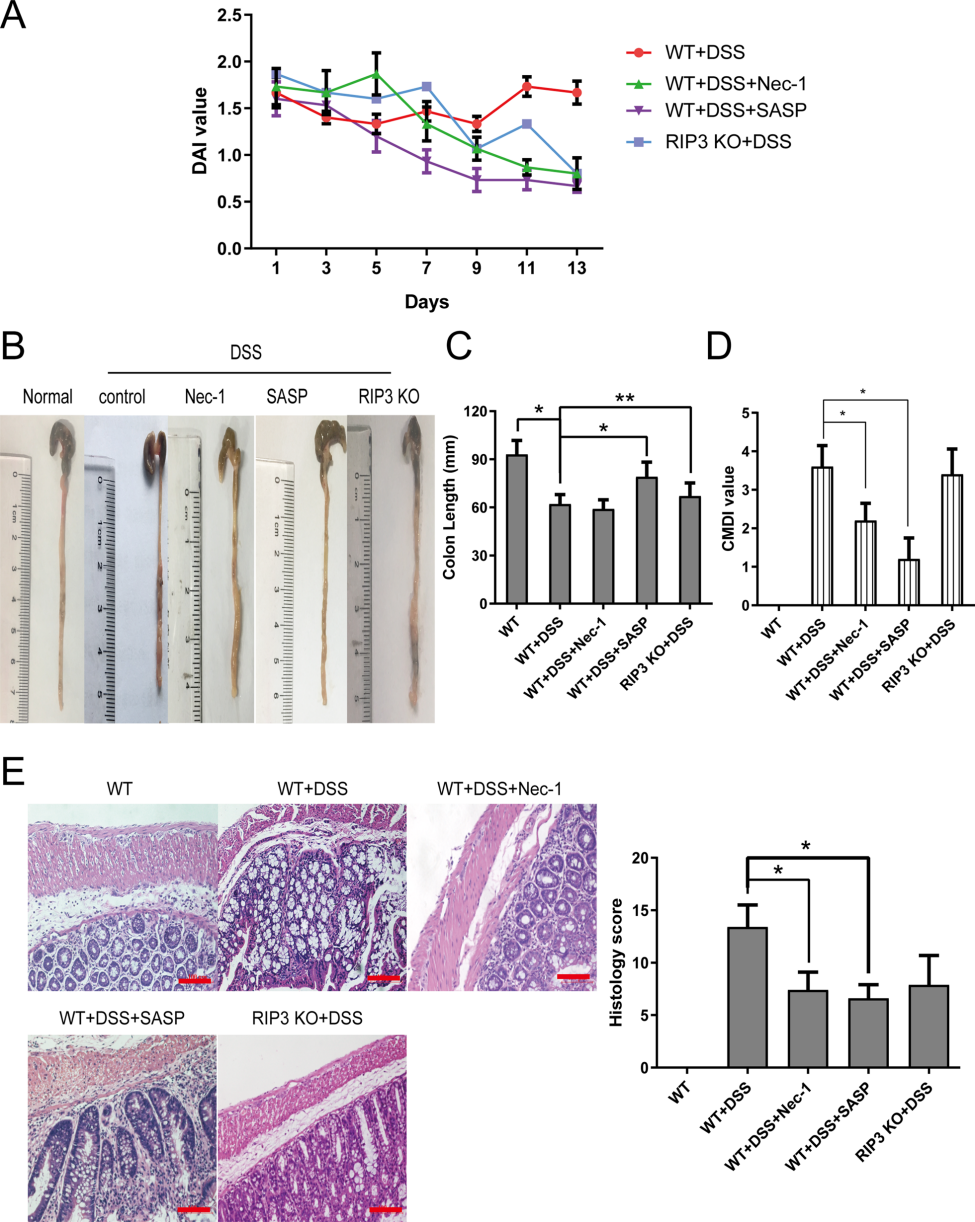
Nec-1, a RIP3 inhibitor, treatment could relieve intestinal pathology and histopathology in DSS-induced colitis mice. **A**–**D** Balb/c wild-type and RIP3 knockout mice freely drank 3% DSS solution for 7 days. On the 8th day, Nec-1 (5 mg/kg), SASP (50 mg/kg) and placebo (1% DMSO) were injected intraperitoneally for 14 consecutive days. **A** DAI, **C** the length of mouse colon and **D** CMDI scores were measured, and **B** the entire colonic intestine from the anus to the end of the cecum was isolated and photographed. **E** H&E staining results were imaged under a 20 × objective. Scale bar: 100 μm. The histology scores are shown on the right panel. The data are presented as mean ± SD. The asterisk indicates a significant difference (*P < 0.05) between groups. Abbreviations: DSS, dextran sulfate sodium; SASP, sulfasalazine; DAI, disease activity index; CMDI, colon gross morphological damage index

Some studies have reported the therapeutic effects of RIP3 knockout and Nec-1 on dextran sulfate sodium (DSS)-induced colitis in mice [15, 23–25]. However, the effect of RIP3 deficiency on inflammation is still controversial [15, 25]. To investigate the effect of RIP3 on intestinal pathology and histopathology in DSS-induced colitis mouse model in vivo, we gave BALB/c wild-type and RIP3 knockout mice 3% DSS solution, freely drinking for 7 days to establish the experimental colitis model. On the 8th day, Nec-1 (5 mg/kg), sulfasalazine (50 mg/kg) and placebo (1% DMSO) were injected intraperitoneally for 14 consecutive days. No mice died during the modeling process. After 24 h of modeling, the model animals began to show loose stools, poor mental state, rough fur, reduced food intake, and weight loss. Compared with the WT + DSS control group, the general state of the mice in the WT + DSS + SASP and WT + DSS + Nec-1 group gradually improved, and the DAI score gradually decreased. Compared with wild-type mice, RIP3 knockout mice gradually recovered from DSS-induced UC after stopping drinking DSS water, (Fig. 4A).

Next, the gross morphology of the colon and CMDI score showed that the surface of the colon of the normal control group was smooth and the texture was clear. In the WT + DSS group, the surface of the intestinal cavity was uneven, and inflammatory congestion and edema were more obvious, accompanied with the enhanced adhesion of intestinal wall to the mesentery. Compared with the WT + DSS group, the intestinal cavity surface congestion and edema in the WT + DSS + SASP group were significantly relieved. The length of the colon was similar to the normal control group, and the CMDI score was significantly reduced (*P* < 0.05). Local congestion and edema were seen in the colon of mice in the WT + DSS + Nec-1 group, mainly in the distal colon, and the CMDI score was significantly decreased (*P* < 0.05) (Fig. 4B–D). The colon of RIP3 KO mice still showed obvious congestion and edema, and the CMDI score was slightly lowered, but it was not statistically significant (Fig. 4B–D). The results of H&E staining indicated that there was no obvious inflammatory cells infiltration in normal control group, and a complete intestinal mucosal structure was visible. The structure of the colon glands in the WT + DSS group was severely damaged, and the glands were arranged disorderly. A large number of lymphocytes and neutrophils infiltrated. Compared with the WT + DSS group, the histopathological changes of the colon in the WT + DSS + SASP group were significantly reduced; the colonic mucosa of the WT + DSS + Nec-1 group was relatively intact with a small amount of inflammatory cell infiltration (Fig. 4E).

### Nec-1 treatment relieves DSS-induced intestinal inflammation in vivo

The balance of intestinal epithelial cell death and proliferation is the basis for maintaining the intestinal mucosal barrier function. Excessive cell death and shedding may cause intestinal barrier dysfunction [26]. The intestinal mucosal barrier is composed of an epithelial cell layer and a mucous layer, which can effectively prevent the invasion of bacteria, toxins and inflammatory mediators in the intestinal lumen. Tight junction (TJ) between intestinal mucosal epithelial cells can prevent harmful substances from penetrating into the submucosa, and the main tight junction proteins include ZO-1 protein [27]. The results of immunofluorescence staining showed that the colon of the model group showed decreased fluorescence intensity of ZO-1compared with the normal control group. Compared with the model control group, the fluorescence intensity of ZO-1 of the WT + DSS + SASP group and the WT + DSS + Nec-1 group increased significantly. However, there was no significant change in the RIP3 KO + DSS group (Additional file 1: Figure S1A).

**Fig. 5:**
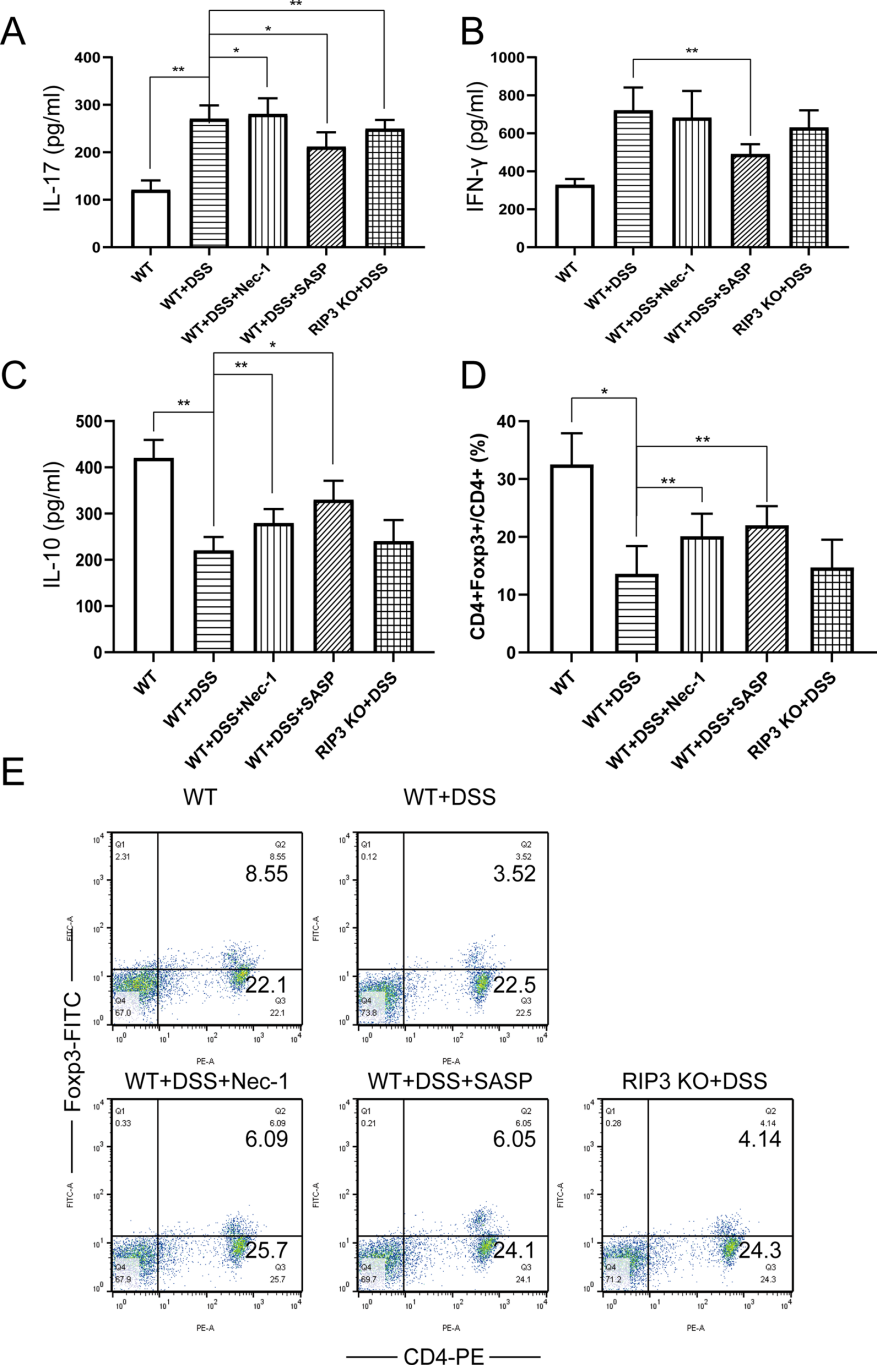
Nec-1 treatment could relieve DSS-induced intestinal inflammation in vivo. **A**–**C** ELISA detected the secretion of IL-17, IFN-γ and IL-10 cytokines in mouse colon tissue. **D**–**E** Flow cytometry analyzed the proportion of CD4^+^ Foxp3^+^ T cells in CD4^+^ T cells of mouse spleen

Altered expression of molecules involved in the necroptotic pathway, causing excessive epithelial cell death, has been shown to trigger intestinal inflammation [7, 28]. Regulatory T cells (Treg) are important for the homeostasis of the immune system. The functional defects of Treg cells or their low proportion in all T cells may lead to inflammatory diseases. Numerous studies have shown that the proportion of Treg cells in peripheral blood mononuclear cells in patients with UC has decreased. Treg has plasticity, such as loss of Foxp3 expression and increased levels of pro-inflammatory cytokine IL-17 and IFN-γ [29, 30]. ELISA results showed that compared with the normal control group, the level of cytokine IL-17 in the colon tissue of the model group was significantly increased, while the level of IL-10 was reduced (*P* < 0.01). Compared with the model control group, IL-17 and IFN-γ levels in the WT + DSS + SASP group were decreased (*P* < 0.01), while IL-10 levels increased (*P* < 0.05). The level of IL-17 in the WT + DSS + Nec-1 group increased slightly (*P* < 0.05), while the level of IL-10 increased significantly (*P* < 0.01) (Fig. 5A–C). Consistently, the level of IL-17 in the RIP3 KO + DSS group decreased slightly (*P* < 0.01). Further, flow cytometry results showed that compared with the blank control group, the ratio of CD4^+^Foxp3^+^ T cells to CD4^+^ T cells in the spleen of the model control group decreased (*P* < 0.01). Compared with the model control group, the proportion of CD4^+^Foxp3^+^ T cells in the spleen of mice in the WT + DSS + SASP group and WT + DSS + Nec-1 group increased significantly, and the difference was statistically significant (*P* < 0.05). The proportion of Treg cells in the RIP3 KO + DSS group was almost unchanged (Fig. 5D-E).

### Knockdown of RIP3 expression inhibits inflammatory signaling pathways in vivo

**Fig. 6:**
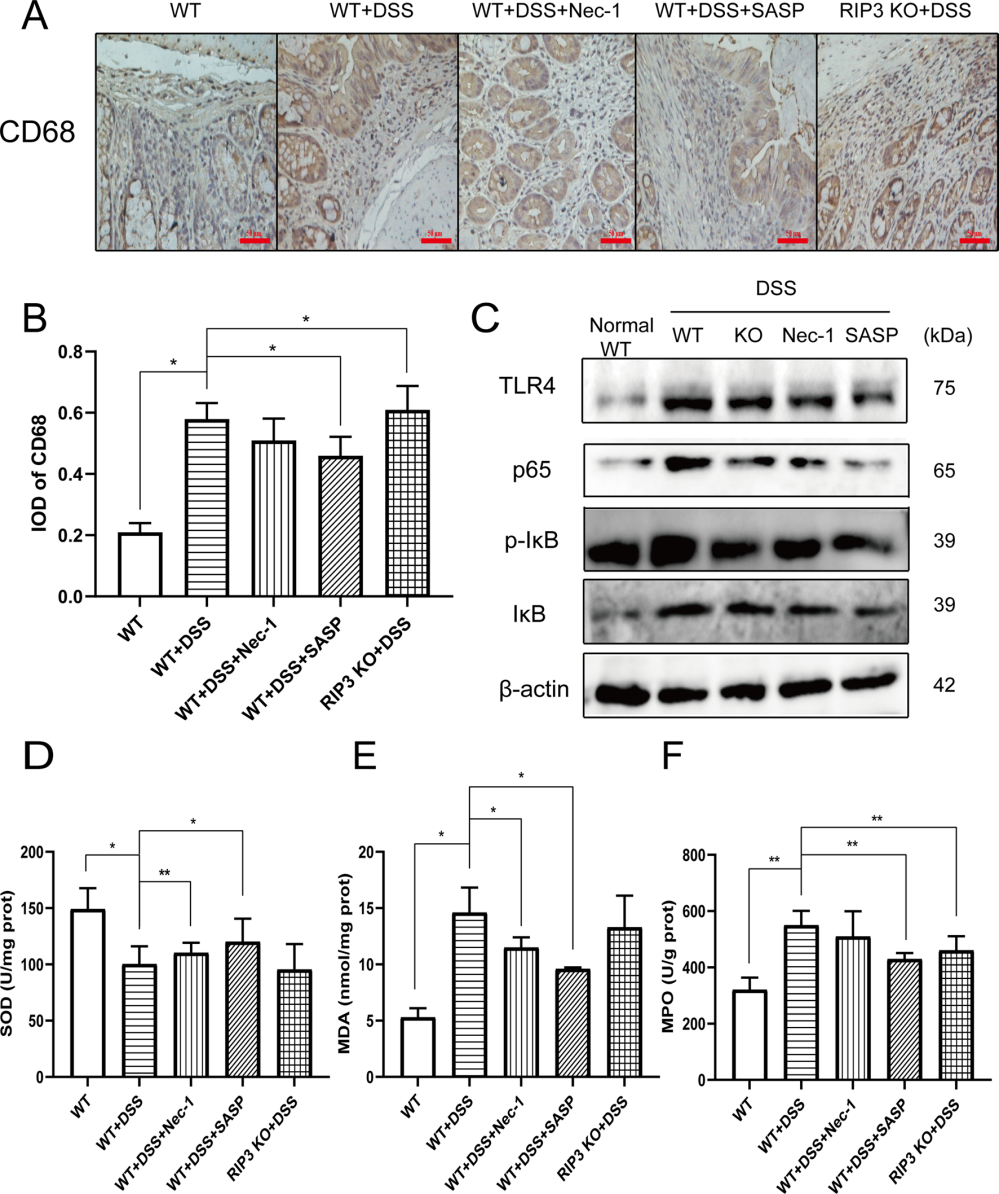
Knockdown of RIP3 expression inhibits inflammatory signaling pathways in vivo. **A** Immunohistochemistry detected the expression and distribution of CD68 in the mouse colon. Scale bar: 50 μm. **B** The mean optical density value (IOD) of CD68 protein in each field was analyzed by Image-Pro Plus 6.0 software. **C** Western blot detected the effect of RIP3 knockout or knockdown in vivo on key molecules of TLR4/NF-κB signaling pathway. **D**–**F** The levels of **D** SOD, **E** MDA and **F** MPO in the mouse colon collected after 7 days’ administration

To understand the role of RIP3 in the regulation of inflammatory signaling pathways and ROS generation in vivo*,* we used IHC to detect the proportion of CD68^+^ cells in the mouse colon. CD68^+^ monocyte/macrophage infiltration is the hallmark for colonic inflammation. Semi-quantitative analysis showed that the proportion of CD68-positive cells in the colon of colitis mice was significantly increased (*P* < 0.05). Compared with the model control group, the proportion of CD68^+^ cells in the colon of the WT + DSS + SASP group decreased (*P* < 0.05), which the proportion of CD68^+^ cells in the WT + DSS + Nec-1 group also decreased, but it was not statistically significant. Consistent with WT + DSS + SASP group, CD68^+^ cells in the RIP3 KO + DSS group was slightly increased (*P* < 0.05) (Fig. 6A–B).

Subsequently, western blot detected the effect of RIP3 knockout or knockdown on key molecules of TLR4/NF-κB signaling pathway in vivo. The results showed that DSS induces the expression of TLR4, p65 and p-IκB in mouse colon tissue. Compared with the model control group, the expression of the TLR4, p65 and p-IκB in the colon of the WT + DSS + SASP group and WT + DSS + Nec-1 group was significantly down-regulated. The levels of p65 and p-IκB in the RIP3 KO + DSS group were significantly down-regulated (Fig. 6C). In order to explore whether RIP3 knockout or knockdown in vivo exerted an anti-apoptotic effect on cells in colon by regulating ROS, we collected the colon tissue of mice 14 days after the administration and tested the levels of SOD, MDA and MPO. The results showed that the SOD level in the colon tissue of the colitis mice was significantly reduced compared with the normal control group (*P* < 0.05), while the MDA and MPO levels were significantly increased (*P* < 0.05). Compared with the model control group, the SOD level in the WT + DSS + SASP group increased significantly (*P* < 0.05), while the levels of MDA and MPO decreased (*P* < 0.05). The SOD level in the WT + DSS + Nec-1 group increased significantly (*P* < 0.01), while the MDA level decreased significantly (*P* < 0.05). Besides, the MPO level in the RIP3 KO + DSS group decreased significantly (*P* < 0.01), suggesting that RIP3 inhibitor Nec-1 can alleviate the oxidative damage of the mouse colon induced by DSS (Fig. 6D–F).

## Discussion

Only by strictly controlling the speed of epithelial cell proliferation and cell death can the integrity of the intestinal structure and an effective intestinal barrier be maintained. Excessive death of intestinal epithelial cells can induce inflammation, leading to human gastrointestinal diseases, including inflammatory bowel disease (IBD). Crohn's disease (CD) and UC are two of the main forms of IBD.

Traditionally, apoptosis and necrosis are the two main forms of cell death, which play a critical role in intestinal epithelial turnover and tissue homeostasis. In recent years, a new mode of programmed cell death that was not related to caspase has been discovered in intestinal epithelial cells, called necroptosis [19]. RIP3 is an important kinase in the cell necroptosis signaling pathway, and it is also related to the antiviral cell necrosis signaling in DNA virus infection. Studies have found that in the intestinal epithelial cells infected by Coxsackie virus, RIP3 can regulate autophagy and aggravate the viral infection of intestinal epithelial cells. RIP3, as an effector protein of the bifurcation point of TNF-α promoting apoptosis and necrosis, plays a key role in the regulation of necroptosis caused by viral bacterial infections, autoimmunity and other reasons [31]. In the study, we collected clinical colon tissues from patients with mild, moderate, and severe UC according to the criterion of the modified Truelove & Witts severity classification and found that the expression of RIP3 in the colon was positively proportional to the severity of UC.

RIP3 inhibitor alleviates pro-inflammatory cytokines production from UC patients PBMCs and splenocytes [32]. RIP3 inhibitor (Nec-1) reduces intestinal inflammation and colitis-associated tumorigenesis in mice [23]. RIP3 inhibitor (GSK872) prevents concanavalin A (ConA)-induced immune-mediated hepatitis (IMH) by reduced hepatic proinflammatory cytokines and immune cells [33]. Losartan inhibits RIP3 expression, causing the inhibition of necroptosis and the alleviation of cardiac hypertrophy. Zhang et al. [34] demonstrated that RIP3 inhibitor (Nec-1) prevents cardiac contractile dysfunction by downregulating the RIP1/RIP3/MLKL signaling pathway. Besides, necroptotic inhibition might be a novel strategy for the treatment of acute myeloid leukemia through the combination of RIP1/RIP3 inhibitor with IFN-γ. Bufalin increases RIP1/RIP3 and ROS, leading to poly(ADP-ribose) polymerase (PARP)-dependent tumor cell death and tumor growth inhibition in human breast cancer cells [35]. However, no clinical trial in progress was related with RIP3-mediated necroptosis.

We successfully established a DSS-induced colitis mouse model. The pictures under a light microscope showed that the glands were arranged disorderly, and a large number of lymphocytes and neutrophils infiltrated the mucosa and submucosa. One week after modeling, mice were treated with Nec-1, a RIP3 inhibitor, for consecutive 14 days. The diarrhea, bloody stool and inflammation of the intestinal mucosa were alleviated significantly. The scores of DAI and gross morphology were significantly lower than those of the model control group. This indicates that RIP3 inhibitors have a good effect on experimental mouse colitis induced by DSS.

RIP3 is involved in tumor necrosis factor receptor (TNFR) signaling, leading to NF-κB-mediated programmed cell death, including apoptosis and necroptosis. In this study, we demonstrated that RIP3 knockdown could suppress TNF-α induced apoptosis. When the pan-caspase inhibitor z-VAD-fmk blocks apoptotic cell death, cells use necroptosis as an alternative cell death pathway [36]. The complex containing RIP3 can be used as a "liposome" to interact with glycogen phosphorylase, glutamate-ammonia ligase and glutamate dehydrogenase 1 after treatment with TNF-α and z-VAD-fmk, and enhance their catalytic activity. The increased activity of these bioenergetic enzymes leads to higher energy metabolism, and subsequently increases the production of reactive oxygen species (ROS) [37, 38]. Further, we tested the levels of oxidants and antioxidants to assess the degree of oxidative stress in mice with colitis. After colitis mice received Nec-1 treatment, their SOD levels increased, and MDA and MPO levels decreased. The results showed that when mice showed oxidative stress damage during colitis, RIP3 inhibitors showed a protective effect.

Inflammation and oxidative stress play a key role in the pathophysiology of UC. External or internal stimuli can activate the inflammatory response, leading to oxidative stress and inflammatory cell infiltration [39]. Necroptosis is considered an "unsafe" method of cell death. Studies have shown that necroptosis can induce the inflammatory response of intestinal epithelial cells and change their cell membrane permeability, while RIP3 can aggravate their inflammatory response and cell membrane permeability [40]. Targeting RIP3 to inhibit necroptosis is possible to reduce the inflammation of the intestinal mucosa. Does RIP3-mediated cell necroptosis play an important role in the pathogenesis of UC? Endogenous ligand/receptor signaling such as vitamin D/Vitamin D receptor (VDR) may inhibit RIP3-mediated necroptosis; Vitamin D and VDR signaling has been shown to have immune-protective effects on inflammatory bowel disease [41] and suppress necroptosis in intestinal epithelial cells by binding RIPK1/3 necrosomes [42]. In this study, we applied two RIP3 inhibitors—Nec-1 and GSK872. They can both inhibit necroptosis and inflammation [43–45]. In our study, sulfasalazine (SASP), a first line therapeutic medicine of IBD, was used as a positive control at the dose of 50 mg/kg to evaluate the efficacy of Necrostatin-1. SASP could attenuate inflammation and reverse DSS-induced activation of RIP3 and MLKL. Thus, SASP protect the intestinal barrier by inhibiting epithelial necroptosis [46].

The TLR4/MyD88/NF-κB pathway is a key factor involved in the inflammatory process [47–49]. The influence of RIP3 on NF-κB activation remains controversial. We further explored the regulatory effects of RIP3 inhibitors or knockdown plasmids on the TLR4/MyD88/NF-κB signaling pathway in human intestinal epithelial cells and mouse colon tissues. The results showed that RIP3 inhibitors and RIP3 knockdown attenuated the release of pro-inflammatory cytokines by inhibiting the activation of TLR4/MyD88/NF-κB, thereby inhibiting the inflammatory response of intestinal epithelial cells induced by TNF-α or DSS in vitro and in vivo. Treg cells are a subset of CD4^+^ T lymphocytes with inhibitory activity and play an important role in controlling the immune response. Due to the lack of immunosuppressive regulation of Treg cells, effector T cells can trigger excessive intestinal immune responses and ultimately lead to intestinal mucosal damage. Treg cells mainly secrete cytokines such as IL-4, IL-10 and TGF-β1 [50]. This study found that Nec-1 can promote the ratio of CD4^+^Foxp3^+^ T cells to CD4^+^ T cells in the mouse spleen, while promoting the expression of IL-10 and inhibiting the expression of IL-17 and IFN-γ. This further indicates that Nec-1 treatment could relieve DSS-induced intestinal inflammation in vivo*.*

RIP3 seems to be the most important promoter of necroptosis. In this study, we only investigated the impact of RIP3 knockdown on inflammation in the colon instead of the small intestine. In the follow-up study, we will further explore the role of RIP3 in patients with CD and mouse models of intestinal inflammation. Besides, further research on necroptosis may include the identification of other RIP1 and RIP3 substrates. Overall, research on RIP3 inhibitors provide important insights for the diagnosis and treatment of necroptosis-related diseases.

## Conclusions

RIP3 knockdown in vitro reverses the inhibitory effect on the proliferation and the stimulative effect on the apoptosis and necrosis of human intestinal epithelial cells induced by TNF-α. Nec-1, RIP3, inhibitor has a therapeutic effect on colitis mice via promoting ZO-1 expression and the proportion of Treg cells, and alleviating the secretion of inflammatory cytokines and necroptosis of mouse intestinal epithelial cells by inhibiting TLR4/MyD88/NF-κB and ROS generation.

## Supplementary Information

**Additional file 1.** The immunofluorescence staining of ZO-1.

## Data Availability

The data that support the findings of this study are available from the corresponding author upon reasonable request.

## Abbreviations

UC: Ulcerative colitits
RIP3: Receptor interacting protein kinase 3
MTT: 3-(4,5-Dimethylthiazol-2-yl)-2,5-diphenyltetrazolium bromide
ROS: Reactive oxygen species
ELISA: Enzyme-linked immunosorbent assay
DAI: Disease activity index
SOD: Superoxide dismutase
MPO: Myeloperoxidase
TNF-α: Tumor necrosis factor-α
ZO-1: Zonula occludens-1
Nec-1: Necrostatin-1
NF-κB: Nuclear factor kappa B
TLR4: Toll-like receptor 4
MyD88: Myeloiddifferentiationfactor88
