# Supplementary material for: RIP3 knockdown inhibits necroptosis of human intestinal epithelial cells via TLR4/MyD88/NF-κB signaling and ameliorates murine colitis
Source: BMC Gastroenterol. 2022 Mar 26;22:137. doi: 10.1186/s12876-022-02208-x (PMC8961930; doi:10.1186/s12876-022-02208-x)
Supplement: Supplementary file 1 — Additional file 1. The immunofluorescence staining of ZO-1. [file 12876_2022_2208_MOESM1_ESM.docx]

**Figure S1**


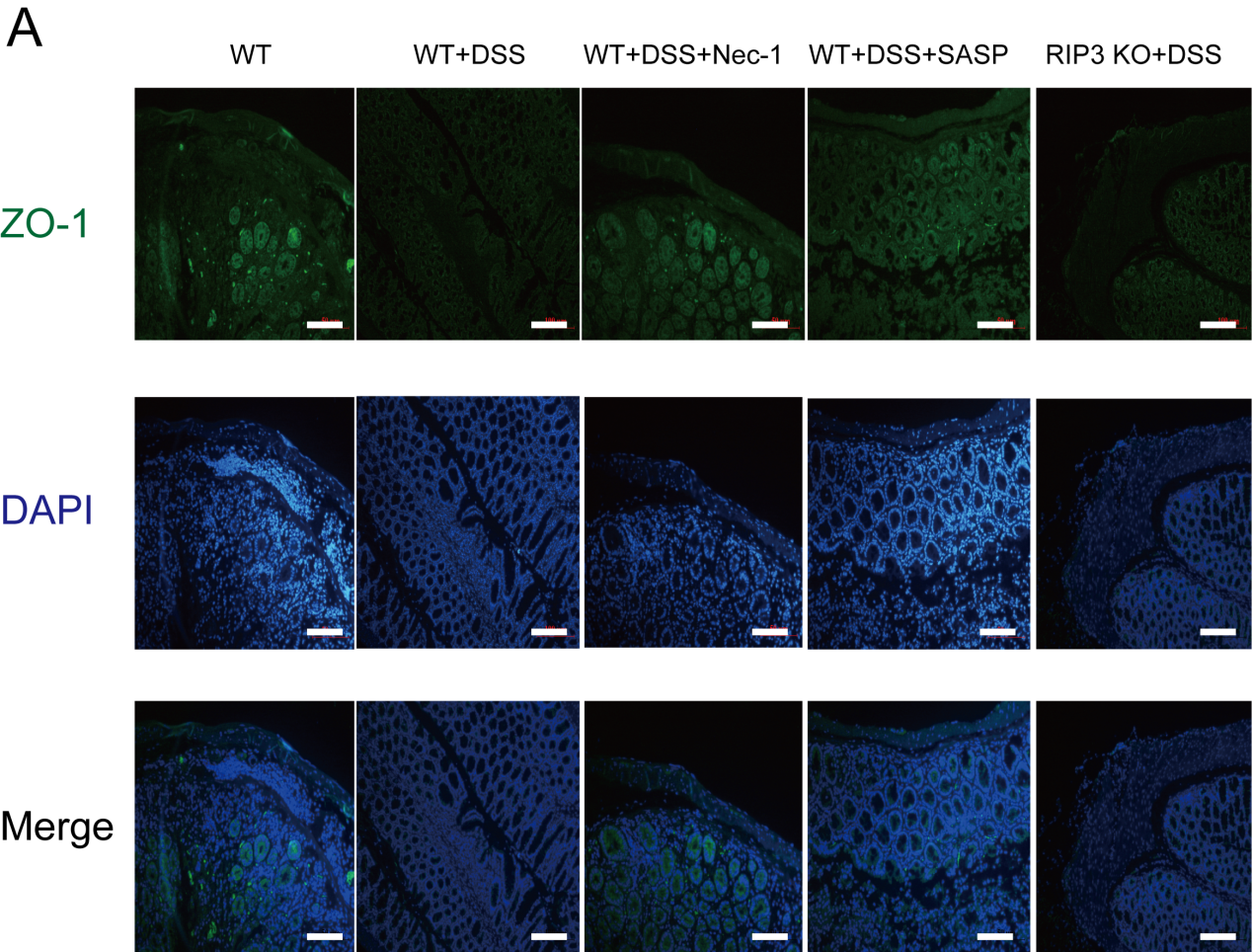


1. Fluorescence microscopy detected the distribution of ZO-1-FITC in mouse colon tissue. Scale bar: 50μm.
